# Dress—From the Health Point

**Published:** 1869-10

**Authors:** B. H Pratt


### Dress—From the Health Point.

BY B. H PRATT, A. M.

Dress—its uses and abuses—how it affects and what it effects—the investing therewith and the divesting thereof—the multiform changes therein and the much talk thereupon—these are universal now; have been ever since our shame-struck mother joined the leaves of fig; will be until the elements, with fervent heat dissolved, shall melt our pride and vanity and love of show, or snatch from us our corporeous forms and make but specters of us.

Wherewithal shall we be clothed? Momentous theme! Can artist fitly paint its hues to youthful eyes? Can sculptor justly model forms so rare? Can poet meetly sing its lays? Never! Then let not any scribe attempt to scribble the æsthetic in domain of Dress, lest such a *role* should roll him on oblivion's rolls.

I do not, then, purpose preposterous things when I essay *hygienic* dress—dress that may not injure, if it do not aid. Hence, not a word from me of cost or quality of what we wear; but of the quantity I may discuss, finding, perchance, that piled too lavishly, it ill assorts with healthful vigor; or, may be, spread out too gingerly, it can adorn, but cannot profit. I like a *show* of use, if nothing more, for every article that man or woman wraps in. What may be beautiful—what the reverse—these are indeed wide fields for wider experience—grand thoughts for grander pens—I'll not offend in these. We dress for health's sake, it is hoped; that is, we mean to. Let's see if we do.

There seems to be a firm fixed folly in the modern mind, that comeliness is not compatible with comfort, in the realm of dress—that one must yield if other enters—that freedom is the foe of form—that loyalty to ease implies base treason to the lines of beauty in the human form divine. Well, that depends. Some cull a clover top with more ecstatic joy than others feel who pluck a damask rose. To me, a bumble-bee is prettier than a wasp. Those ladies who affect the latter, it seems to me, lose time, if nothing else. By not affecting, they'd possess the prettier form. Nature says so, but she's a dunce to Art. So goes l'monde. But who are they that mar their forms and cry a graceful act performed?

The human male is scarcely open to attack in this especial line, although there be isolated instances of unmanly folly among us in the way of be-laced and be-padded masculinity. That their models of the beautiful are correct, or that their brains are normal, are both questionable, to say the least. Perhaps such have their uses here below—are not created in vain though vain creations—need no advice, since health is of no especial moment to themselves or others. Strange phantasy for men! I never knew a portly man, whose portliness did not rather grace than disfigure him.

But there are those whom we pity and would aid—who pine under an affliction we would alleviate—who writhe in a clutch we would release—who sink beneath a burden we would lift—who die piecemeal, either too fearful of Madame Mode to defy her, or too ignorant of a remedy to invoke it.

Female dress, to-day, is suicidal. My dear Miss—my honored Madam—let me say to you a few plain, well-meaning words. Do not faint. I don't mean to draw blood nor wrench a muscle. Every inch you contract your waist beyond nature's limit, shortens your lungs by the same measure. Shortening the lungs one inch, lessens its capacity for receiving air, the life-giving constituent, just one half pint. Have you lungs to spare, when consumption rides Argus eyed in search of pulmonary defects? Do you desire to forfeit for a lifetime the privilege of inhaling the measure of four ounces of pure air at each inspiration—one gallon per minute? Can you afford to barter so much life for so little questionable beauty? If one inch costs so dear, what will six, ten, twelve, (all within the limits of many of you) cost? The estimate, you see, if carried out, becomes something fearful.

"But you are not conscious of over-pressing your waist." Custom has insensibly deprived you of pain from abdominal pressure. Would you hear a cry of agony? girt up to your own attenuations the yet unspoiled country lass, the child of nature, of your own weight and height. Do you ever realize what you do at every pull you give your corset-strings? You compress your abdominal muscles—deprive them of nourishment—that is, deform. You impede the circulation of blood—that is, paralyze. You flatten the stomach—cause indigestion. You disturb the liver—induce biliary affections. You force up the diaphragm—distort. You shorten the lungs—court consumption. You compress the heart—artificialize the pulsations. Depress the intestines—bowel difficulties. You derange the organs in the lower part of the abdomen—urinary difficulties—lapsus uteri—multiform female weaknesses. Is *suicidal* too strong a term to apply to tight, or even moderate lacing?

But, you not only lace—you pad. You smother with a non-conducting material from one to four inches in thickness, that portion of the body for which both nature and materinty clearly demand comparative freedom. You do this, knowing that you are detracting, day by day, from your usefulness as a mother—actually depriving your present or prospective offspring of its natural sustenance, and superinducing the mammary difficulties and diseases so fearfully prevalent in our own day. I ask you, plainly and compassionately, (with fear of offending a modesty I sincerely honor, yet incapable of withholding from you facts of so vital an importance,) to elevate yourselves above these follies of fashion and destroyers of life—above these sure disseminators of disease and debility among the coming generations—and consult Nature oftener than Art—Health oftener than Fashion—the good of the race oftener than present vanity. Let history warn you. Show me where luxury has pampered a nation into follies, similar, if not like, ours, and I will show you a degenerate race, without power to resist the inroads of a once despised invader.

Good men have too long bewailed in silence the danger impending over the generations to come, ushered into the world with a worm gnawing at their vitals. Yet they whose duty it is to proclaim to our Mothers and Wives and Sisters and Daughters the pernicious effects of society's so-called claims, refrain from the task—shirk the duty incumbent upon them—avoid the thankless, nay, invidious work.

Would that truth were not so hateful to hear—that men did not prefer to fling the darts of ridicule and hurl the shafts of sarcasm at follies so gigantic in their magnitude—so momentous in their sway—so disastrous in their effects—so universally prevalent as to threaten the health of every woman—the happiness of every home—the stability of the nation itself. Shall the vanity of woman plunge us into effeminacy? Why is truth, plainly pointed and practically aimed at actual error, so repulsive to the mass of man and womankind; while fiction, reeking with the glut of shame and degradation scarce conceivable, lies open upon our news counters and centre tables, ever at hand to poison an idle hour? Tell woman all your glowing imagination can picture of the errors, the sins, the crimes, the crimson guilt of her sex, and she will lavish honors upon you *ad nauseam;* but breathe to her one word of the horrible truth she daily revels in, and you and the sheet you print become things too vile to be countenanced by a so-called respectable community. "He lacks dignity!" Indeed? Dignity! and the epithet from *such* a source! The day is coming, however, when Medicus must preach as well as practice, else the limit of self-indulgence will be found in self-destruction. The medical world needs some such philanthropist—some such crier of abuse therein, as the dramatic world has just ushered upon us in the person of Olive Logan; who believes in calling things by no mincing, kid-glove names, nor hesitates to point at and stir up any dirty mess that bids fair to foul the drama she defends. She compels a hearing, too. But we are not through.

A matter of abuse in dress, though less formidable than those just mentioned, strikes me as wofully detrimental to feminine health. It is not a question—it will not stand debate—as to whether woman is properly, protectingly clad from the waist downward. Reason affords no affirmative argument. That woman is as healthful as she seems, is one of the enigmas of the day, when we consider to what changes of temperature her limbs are subjected. From the heated apartment to the frosty street air, and but a single thickness of cotton cloth to temper the force of the chilling blast! To what torture can the human body not submit when it persists in suffering.

Consumption again finds a powerful adjunct in a habit quite common among the thoughtless ones. When Autumn approaches, our young ladies, with prudent forethought and profound reliance upon their constancy to healthful duty, don the vest or wrapper or other chest-protecting under-garment, with full determination to wear the same during the cold weather. But the first invitation to a fashionable evening out tempts them successfully to discard the wrapper for the nonce, since Fashion, forsooth, imperious dame that she is, may have decreed to honor only those who may bare their necks to her inquisitorial eye. Do we need to wonder, then, at the oft-complained of cold, catarrh, or bronchial difficulty on the part of those who thus deliberately discard a protector when most needed? Need we look further for cause of hacks that worry fond mammas, and coughs that horrify the household?

Are our modern belles wholly innocent of shoes with soles that rival paper in their thinness?—of bonnets that are such in name but not in fact? Are muffled hands, and cuffed wrists, and furred necks quite consistent with gauze-covered ankles in December?

I might proceed *ad infinitum*, but I close with only the above open questions to your own sense of self-justice. May you whom they interrogate entertain them honestly, in the quiet of your chamber, unprejudiced by fashion's follies, and act your reply in all coming toilets.

